# Multiple levels of the unknown in microbiome research

**DOI:** 10.1186/s12915-019-0667-z

**Published:** 2019-06-12

**Authors:** Andrew Maltez Thomas, Nicola Segata

**Affiliations:** Department CIBIO, University of Trento, Trento, Italy

## Abstract

Metagenomics allows exploration of aspects of a microbial community that were inaccessible by cultivation-based approaches targeting single microbes. Many new microbial taxa and genes have been discovered using metagenomics, but different kinds of “unknowns” still remain in a microbiome experiment. We discuss here whether and how it is possible to deal with them.

Our understanding of the microbial communities that inhabit the human body and other environments has greatly improved in the past decade due to both biotechnological and computational advances in the metagenomic field [1]. Of particular note are the successful efforts to identify and genetically describe new microbial species that were previously part of the set of unknown micro-organisms occasionally referred to as “microbial dark matter”. However, in a typical microbiome experiment, several aspects of microbial communities still remain inaccessible. This inability to fully explore the diversity of a microbiome in a sample occurs at multiple distinct levels (Fig. 1) and should be acknowledged to avoid mis- and over-interpretation.

**Fig. 1.**
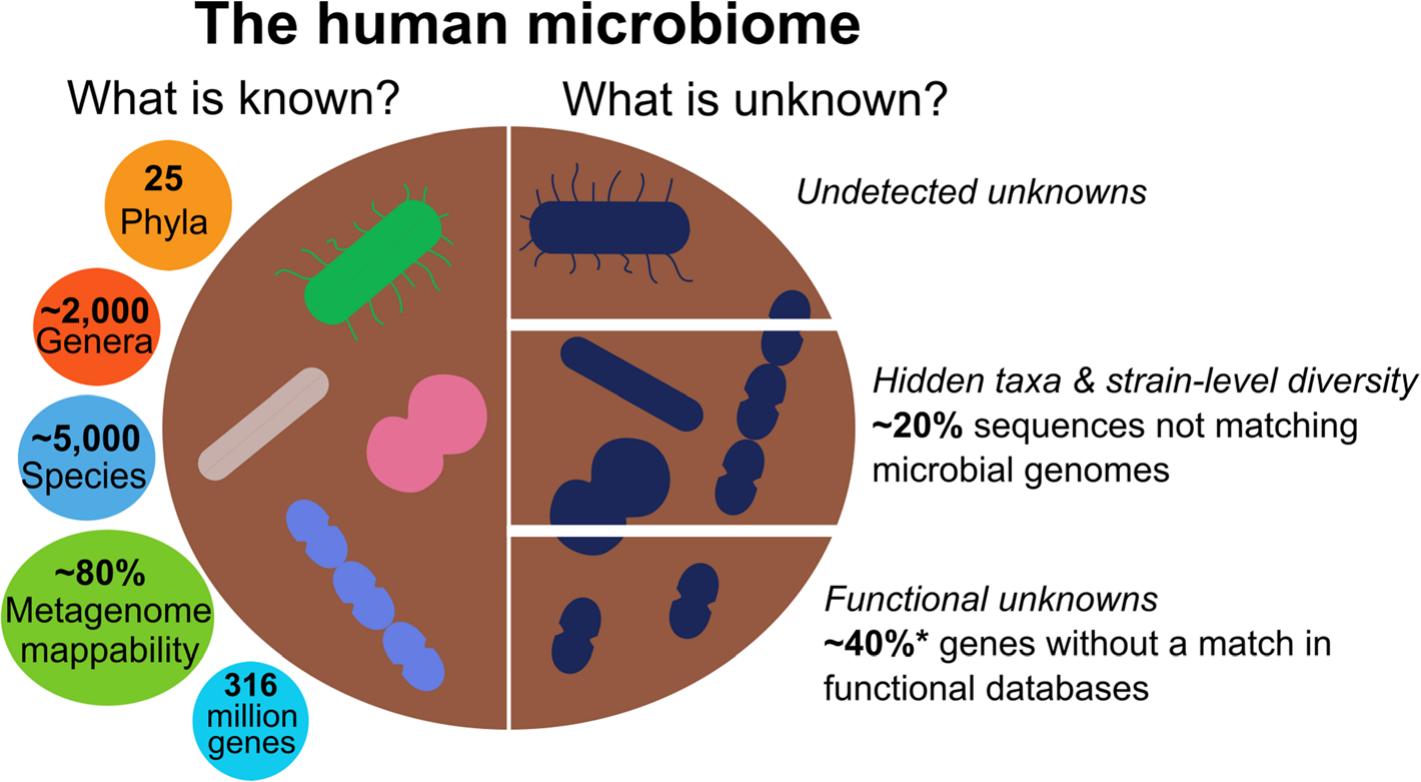
The current knowns and unknowns in the human microbiome. Numbers of known and unknown members of the human gut microbiome taken from a population-wide and multi-bodysite large-scale metagenomic assembly study [2]. Numbers marked with *asterisks* refer to genes from the Integrated Gene Catalogue (IGC) of the human gut microbiome and are derived from human fecal samples and mapping to the eggNOG database [3]

At the deepest level of hidden diversity there are those members of the community that are not captured at all by the experiment, the *undetected unknowns*. These include low-abundance but potentially crucial taxa, whose genetic material is not sampled by sequencing techniques due to being present below the level of detection. Exactly where this threshold lies depends in part on experimental choices and specific techniques; for example, the dominance of host cells and DNA in the sample (e.g., biopsies from the intestinal mucosa) makes microbial taxa harder to detect and is a common problem in metagenomics experiments. Cultivation is less sensitive to the microbial concentrations in the sample than sequencing-based approaches and has contributed significantly to characterizing low-abundance taxa, especially when applied in a high-throughput setting [4]. However, available isolation protocols are unavoidably biased towards certain classes of microbes and are successful only for a fraction of a microbiome’s biodiversity. Bacteriophages are particularly prone to being under-sampled due to their short genomes and biochemical properties (e.g., having an RNA or single-stranded DNA genome) that are typically not considered by standard sample preparation protocols. Although virome enrichment protocols have been developed and applied, viruses remain perhaps the most neglected class of members of microbial communities.

Microbiome taxa whose DNA is at least partially sequenced in the microbiome experiment but have not been described before and are phylogenetically far from genomes deposited in public databases represent another level of uncharacterized diversity. It was for such hard-to-profile *hidden taxa* that the term “microbial dark matter”, inspired by physics, was initially coined [5, 6]. This analogy has, however, come under question [7], since the dark matter in physics is thought to be a different form of matter while in microbiology undiscovered microbes have the same molecular basis as the known ones. This type of microbial hidden diversity is efficiently targeted by large-scale isolate sequencing and metagenomic assembly efforts that have recently uncovered many previously unexplored taxa [2, 8]. As a result of integrating the new taxa in the set of reference genomes, microbiomes can then be more comprehensively analyzed because the fraction of reads from a shotgun sequencing experiment that match a catalogued microbial genome—i.e., the metagenome’s mappability—increases. Our knowledge of the overall diversity of the human gut microbiome has indeed been greatly improved by large-scale metagenomic assembly efforts. For example in our study [2], mappability rates of gut metagenomes reach averages above 85% (median close to 95%), while previous rates were in the 50–70% range. Independent efforts based on both metagenomics [9, 10] and large-scale cultivation [8] have confirmed this trend. The mappability of metagenomes from human body sites other than the gut, such as the skin and the oral cavity, was similarly increased [2], and also for more diverse non-human environments these approaches have proven to be efficient and promising [11]. However, organizing large numbers of draft genomes from uncharacterized taxa is challenging, and while performing well for bacteria, assembly-based metagenomic tools are less effective when targeting new eukaryotic microbes and viruses.

Intra-species genomic diversity can be extensive in bacteria and archaea. Indeed, several isolate-sequencing studies on (potential) pathogens highlighted how the set of genes that are present in some but not all the strains of a given species (i.e., the accessory or variable genome) can be more than ten times larger than the set of “core” genes that are always present in all strains of the species. Because the majority of microbiome species have few (if any) available genomes, the accessory genome of many species is underrepresented and thus the fraction of unmappable genetic material in a microbiome belonging to regions other than the core genome can be extensive. This is highlighted by the ~ 8% increased mappability that was observed when gut metagenomes are aligned against all > 154,000 newly recovered metagenomically assembled genomes rather than the 4930 single genome representatives of each candidate species (both known and newly defined). This increase ranged from 1.7% in vaginal samples to 23.8% in stool samples from non-Westernized populations [2]. To make further progress in uncovering *hidden strain-level diversity*, it is thus crucial to reconstruct sample-specific assemblies from the analyzed metagenomes and to include as many genomes as possible for each species in reference databases. Because species have pangenomes that are likely to be “open” (i.e., without an upper bound on the size of the accessory genome) mostly due to extensive horizontal gene transfer, it seems technically impossible to recover all strain-level diversity of a species across samples, but continuing the effort of cataloguing strain variants remains crucial for an in-depth understanding of the functional potential of a microbiome.

The functional potential encoded in the overall microbiome and in its single microbial constituents is key to the understanding of microbial communities. The *functional unknowns* of a microbiome are, however, much more extensive and difficult to tackle than their taxonomic counterpart. This inaccessibility to functions stems from our limited understanding of the genes and pathways in a microbial genome, especially for non-model organisms, and from the wide phylogenetic diversity of microbiome members causing sequence homology to only partially capture functional similarity. Functional- and gene-centric efforts to characterize metagenomes include the creation of the Integrated Gene Catalogue (IGC) of the human gut microbiome, which comprises almost 10 million genes [3]. This is a non-redundant resource grouping genes at an identity threshold of ≥ 95% with ≥ 90% overlap, thus collapsing into gene-families the otherwise extremely large set of unique genes in the human microbiome (more than 316 million) [2]. Interestingly, 39.6% of genes present in the IGC catalogue were unmapped to functional databases. And the ability to match a gene against a target in functional databases is, however, only a partial step towards annotating its function; for instance, out of the 60.4% of genes that were annotated in the IGC, 15–20% are genes that have been observed before but are labeled as “function unknown” [3]. These numbers demonstrate how little is still known regarding both the genes that are present in microbial communities and their function. And whereas for taxonomic and phylogenetic diversity the latest high-throughput techniques are quickly decreasing the fraction of inaccessible taxa, experimental functional characterization of genes is inherently difficult to scale in high-throughput and cost-effective systems and is not receiving sufficient research investments. Although comparative analysis of the functional potential of metagenomes in different conditions can help in prioritizing genes for experimental functional characterization, it is very likely that the functional understanding of microbiomes cannot substantially improve in the short term and this appears to be one of the main limiting factors in the field.

Current and future efforts to uncover the unexplored aspects of microbiomes will have direct consequences on several applications. Fecal microbiome transplantation is one such example, as a more complete profiling of gut microbiome samples can allow better and safer selection of donor samples and an improved understanding of which taxa contribute the most to the success of this medical practice. Uncovering the currently inaccessible microbiome members can also be crucial to expand disease-predictive taxonomic and functional microbiome signatures [12], and to better characterize populations and environments that are less studied and thus exhibit larger fractions of unexplored diversity. Several new phyla with intriguing phylogenetic placement in the whole tree-of-life have been recently described using metagenomics [13], and such continued expansion of the catalogued microbial diversity may also aid in our understanding of several biological aspects, including, for example, the process of eukaryogenesis, the origin of the eukaryotic cell [14].

The microbiome field is ready to embrace new and improved technologies to continue current efforts of reducing the effect of the different levels of unknowns in a microbiome experiment. These range from high-throughput cultivation [4] to single cell sequencing [6], but also improved computational methods are needed to more deeply explore metagenomic datasets, especially at a large scale. Functional understanding of the microbiome remains, however, the biggest challenge, and although low-throughput experiments targeting specific genes are irreplaceable, technology can again provide complementary solutions. These include integrated high-throughput profiling of the microbial transcriptome, metabolome, and proteome, and the automation of cultivation-based assays to scale-up the screening of multiple taxa and genes for phenotypes of interest. There are thus the conditions to substantially uncover the currently inaccessible microbiome, but specific differences and challenges are connected with each of the different kinds of the unknown outlined here.

## Data Availability

Not applicable.
